# Cross-sectional associations of nighttime sleep and daytime nap duration with myopia in preschool children: the mediating role of body mass index

**DOI:** 10.3389/fmed.2026.1840892

**Published:** 2026-07-07

**Authors:** Xiumei Chen, Dan Liu, Wei Du, Changhua Wu

**Affiliations:** 1 Department of Child Health Care, Shaoxing Maternity and Child Health Care Hospital, Maternity and Child Health Care Hospital Affiliated to Shaoxing University, Shaoxing, Zhejiang, China; 2 Department of Ophthalmology, Shaoxing Maternity and Child Health Care Hospital, Maternity and Child Health Care Hospital Affiliated to Shaoxing University, Shaoxing, Zhejiang, China

**Keywords:** body mass index, daytime nap duration, myopia, nighttime sleep duration, preschool children

## Abstract

**Objective:**

To investigate the associations of nighttime sleep and daytime nap duration with myopia in preschool children, and to examine the mediating role of body mass index (BMI).

**Methods:**

This cross-sectional study included 695 children aged 3–7 years. Sleep duration, demographics, family factors, and lifestyle were assessed via a self-administered questionnaire. Ophthalmic examinations followed national myopia screening guidelines. Multivariate logistic regression, restricted cubic splines, and mediation analysis were used to evaluate the associations and the mediating effect of BMI.

**Results:**

The prevalence of myopia was 21.29%. After adjusting for confounders, both daytime nap duration [OR = 0.87, (*p* = 0.012)] and nighttime sleep duration [OR = 0.48, (*p* = 0.031)] were negatively associated with myopia risk. A protective inflection point was identified at 38 min for daytime napping. BMI partially mediated the associations of daytime nap and nighttime sleep with myopia, accounting for 24.79 and 28.16% of the total effects, respectively.

**Conclusion:**

Both nighttime sleep and daytime nap duration were inversely associated with myopia risk in preschool children, with BMI playing a partial mediating role. Ensuring adequate nighttime sleep and appropriate daytime napping may represent an effective strategy for early myopia prevention.

## 1 Introduction

Myopia, the most common form of refractive error, has emerged as a major global public health concern (1). By 2050, its prevalence is expected to affect nearly 5 billion people, with high myopia accounting for 1 billion cases (2). High myopia carries a high risk of irreversible blinding complications—such as glaucoma, retinal detachment, and macular degeneration—imposing substantial health and economic burdens on individuals and society (3). In recent years, myopia has shown a trend toward earlier onset, raising concern about its occurrence in preschool children. A survey across 10 provinces in China reported a myopia prevalence of 5.5% among children aged 5–6 years, while the prevalence of pre-myopia reached 37.9% (4), suggesting that the preschool period may represent a critical window for myopia prevention.

Environmental factors play a crucial role in the onset and progression of myopia in children. The International Myopia Institute has identified insufficient outdoor time and excessive near work as major risk factors for acquired myopia (5). Meanwhile, sleep has emerged as a modifiable factor of growing interest in relation to myopia. Existing studies suggest that meeting the recommended sleep duration is associated with a significantly lower risk of myopia (6), whereas insufficient sleep and delayed bedtime may increase the risk (7, 8). For instance, one birth cohort study found that insufficient sleep at 5.5 years of age was associated with increased axial length, and social jetlag of ≥1 h at age 4 also raised the risk of myopia (9). Similarly, the French EDEN cohort study showed that sleep duration and timing at age 2 could influence refractive status at age 5 (10). Animal and clinical studies further suggest that circadian disruption may contribute to myopia development by interfering with the rhythmic changes in axial length and choroidal thickness (11, 12). Nevertheless, findings on the association between sleep duration and myopia remain inconsistent. A systematic review noted that although longer sleep duration was associated with a lower risk of myopia (OR = 0.67 for the highest vs. lowest category), the protective effect did not reach statistical significance across any level of exposure in the continuous dose–response analysis (8). Another study also failed to find a significant association between sleep duration and myopia prevalence (OR = 0.905, 95% CI: 0.782–1.047) (13). These discrepancies may be attributable to differences in sample age composition, sleep measurement methods, and adjustment for confounding factors across studies.

Given that existing research has largely focused on school-aged children, while studies examining the effects of both daytime napping and nighttime sleep on myopia in preschool children remain limited, this study aimed to investigate the associations of these two types of sleep duration with myopia and to further analyze the mediating role of body mass index (BMI), with a view to providing scientific evidence for myopia prevention and control in preschool children.

## 2 Materials and methods

### 2.1 Patients

**Figure 1:**
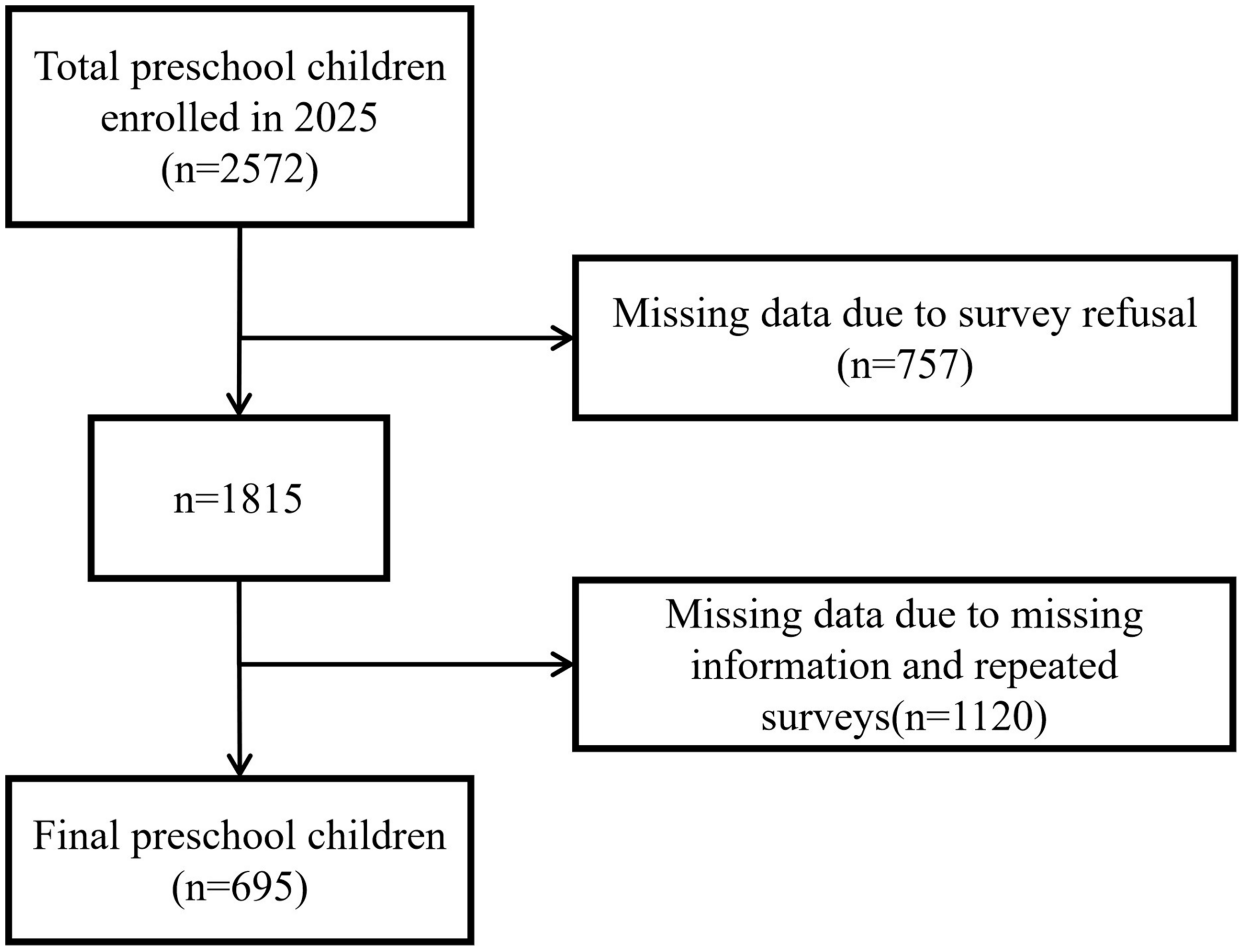
Flowchart of study participants.

This cross-sectional study used a convenience sampling method to select participants. The study was conducted from January 2025 to December 2025, and the participants were preschool children attending the Shaoxing Maternal and Child Health Hospital. Inclusion criteria were: age 3–7 years, enrolled in kindergarten, normal cognitive function, and informed consent provided by guardians. Exclusion criteria included: ocular trauma, corneal diseases, or other non-myopic organic eye diseases (defined as structural eye diseases not related to refractive errors, such as congenital cataracts, glaucoma, or retinal pathologies); and missing key information in the questionnaire. A total of 2,572 eligible participants were initially enrolled. After excluding 1,877 children due to refusal (*n* = 757), missing visual acuity or demographic data (*n* = 1,007), or duplicate surveys (*n* = 113), 695 participants were included in the final analysis (Figure 1). The study protocol was approved by the Ethics Committee of Shaoxing Maternity and Child Health Care Hospital (No. SXSFYBJY-2026028) and adhered to the principles of the Declaration of Helsinki. Written informed consents were acquired from guardians of all participating children before recruitment into the study.

### 2.2 Questionnaire survey

A self-designed questionnaire was used to collect demographic, family, and lifestyle information, developed based on the pediatric myopia risk factor framework from the International Myopia Institute and large-scale epidemiological studies (5, 14, 15). The selection of variables was guided by published evidence: parental myopia and education level were included as genetic and socioeconomic factors (1, 14); sleep duration and timing as modifiable biological factors (6, 9, 14); outdoor activity and near work as environmental factors (5, 16); and BMI as a potential metabolic mediator (17, 18). The complete questionnaire is provided in Supplementary material S1.

Demographic variables included sex, age, BMI, and grade. Age was dichotomized by the median (< 4.80 years vs. ≥ 4.80 years), BMI was divided into tertiles (< 16.7 kg/m^2^, 16.7–17.8 kg/m^2^, and ≥ 17.8 kg/m^2^), and grade was classified as low, middle, or high. Family factors included household registration (urban vs. rural), monthly household income (< 5,000 CNY, 5,000–10,000 CNY, 10,001–15,000 CNY, > 15,000 CNY), marital status (married vs. divorced), paternal and maternal education (junior high school or below, senior high school/vocational school, college or above), and paternal and maternal myopia (yes vs. no). Lifestyle factors included sleep duration (daytime nap and nighttime sleep), daily outdoor activity time (< 1 h vs. ≥ 1 h), daily mobile phone use time (0 h, < 0.5 h, ≥ 0.5 h), daily television viewing time (0 h, < 1 h, ≥ 1 h), and daily after-school study time (0 h, < 1 h, ≥ 1 h). Questionnaires were distributed on site and completed jointly by parents and children, one per participant.

### 2.3 Myopia screening

Ophthalmic examinations were conducted at the hospital by uniformly trained ophthalmologists. Visual acuity testing was performed in accordance with the Specifications for Myopia Screening in Children and Adolescents issued by the National Health Commission and seven other ministries in 2018 (14). Participants first underwent distance visual acuity testing using a standard logarithmic visual acuity chart conforming to the national standard (GB 11533), with a testing distance of 5 meters (15). For those with uncorrected visual acuity below 5.0, after excluding active ocular lesions via slit-lamp examination, refractive error was measured using an autorefractor under non-cycloplegic conditions. Each participant was measured twice by the same optometrist, and the average value was used as the final result. The spherical equivalent (SE) was calculated as sphere + 1/2 cylinder. Myopia was defined as uncorrected visual acuity < 5.0 and non-cycloplegic SE < −0.50 D.

### 2.4 Statistical analysis

Data were analyzed using SPSS version 24.0 and R version 4.2.3. Continuous variables were expressed as mean ± standard deviation or median (interquartile range), while categorical variables were presented as frequencies (percentages). Group comparisons were performed using the t-test, Mann–Whitney U test, or chi-square test, as appropriate. Multivariate binary logistic regression was employed to assess the associations of daytime nap duration and nighttime sleep duration with myopia. Three models were constructed sequentially: an unadjusted model (Model 1), a model adjusted for demographic and family factors (Model 2: sex, age, BMI, grade, family income, marital status, paternal education level, maternal education level, residence, paternal myopia, maternal myopia), and a fully adjusted model with further adjustment for daily eye-use and lifestyle factors (Model 3: Model 2 plus daily outdoor activity time, daily mobile phone use time, daily television viewing time, daily after-school study time). Odds ratios (ORs) and their 95% confidence intervals (CIs) were calculated. Restricted cubic splines (RCS) and threshold effect analysis were used to explore the dose–response relationships and potential inflection points between sleep duration and myopia. Subgroup analyses were further performed stratified by demographic, family, and lifestyle factors, and interaction terms were introduced to evaluate effect modification. Mediation analysis was conducted using the ‘mediation’ package in R, with daytime nap duration and nighttime sleep duration as independent variables, BMI as the mediator, and myopia as the dependent variable. The average causal mediation effect (ACME) and average direct effect (ADE) were estimated, and the proportion mediated was calculated. All tests were two-tailed, and statistical significance was set at *p* < 0.05.

## 3 Results

### 3.1 Participant characteristics

The baseline characteristics of the participants are shown in Table 1. Among the 695 preschool children, 148 had myopia, yielding a prevalence of 21.29%. Compared with non-myopic children, those with myopia had a higher proportion of being older (≥4.8 years), female, from higher-income families, with divorced parents, with parental education at junior high school or below, living in rural areas, with daily outdoor activity <1 h, daily mobile phone use ≥0.5 h, daily television viewing ≥1 h, daily after-school study time of 0 h, nighttime sleep duration ≤8 h, and daytime nap duration ≤1 h (all *p* < 0.05).

**Table 1:** Characteristics of the study participants.

Variables	Total (*n* = 695)	Non-myopia (*n* = 547)	Myopia (*n* = 148)	Statistic	*P*
Age, *n* (%)				χ^2^ = 3.99	0.046
< 4.8 years	299 (43.02)	246 (44.97)	53 (35.81)		
≥ 4.8 years	396 (56.98)	301 (55.03)	95 (64.19)		
Gender, *n* (%)				χ^2^ = 14.48	<0.001
Female	345 (49.64)	251 (45.89)	94 (63.51)		
Male	350 (50.36)	296 (54.11)	54 (36.49)		
BMI, *n* (%)				χ^2^ = 144.83	<0.001
< 16.7 Kg/m^2^	228 (32.81)	228 (41.68)	0 (0.00)		
16.7–17.8 Kg/m^2^	231 (33.24)	190 (34.73)	41 (27.70)		
≥ 17.8Kg/m^2^	236 (33.96)	129 (23.58)	107 (72.30)		
Family income, *n* (%)				χ^2^ = 55.91	<0.001
Low	127 (18.27)	114 (20.84)	13 (8.78)		
Lower-middle	244 (35.11)	211 (38.57)	33 (22.30)		
Middle-high	215 (30.94)	162 (29.62)	53 (35.81)		
High	109 (15.68)	60 (10.97)	49 (33.11)		
Marital status, *n* (%)				χ^2^ = 174.45	<0.001
Married	451 (64.89)	423 (77.33)	28 (18.92)		
Divorced	244 (35.11)	124 (22.67)	120 (81.08)		
Paternal education level, *n* (%)				χ^2^ = 87.73	<0.001
Junior high school or below	204 (29.35)	128 (23.40)	76 (51.35)		
Senior high school or technical secondary school	329 (47.34)	309 (56.49)	20 (13.51)		
College or above	162 (23.31)	110 (20.11)	52 (35.14)		
Maternal education level, *n* (%)				χ^2^ = 67.50	<0.001
Junior high school or below	209 (30.07)	142 (25.96)	67 (45.27)		
Senior high school or technical secondary school	315 (45.32)	292 (53.38)	23 (15.54)		
College or above	171 (24.60)	113 (20.66)	58 (39.19)		
Grade, *n* (%)				χ^2^ = 59.78	<0.001
Junior class	147 (21.15)	115 (21.02)	32 (21.62)		
Middle class	348 (50.07)	310 (56.67)	38 (25.68)		
Senior class	200 (28.78)	122 (22.30)	78 (52.70)		
Residence, *n* (%)				χ^2^ = 93.99	<0.001
Urban	427 (61.44)	387 (70.75)	40 (27.03)		
Rural	268 (38.56)	160 (29.25)	108 (72.97)		
Paternal myopia, *n* (%)				χ^2^ = 76.38	<0.001
No	431 (62.01)	385 (70.38)	46 (31.08)		
Yes	264 (37.99)	162 (29.62)	102 (68.92)		
Maternal myopia, *n* (%)				χ^2^ = 51.70	<0.001
No	451 (64.89)	392 (71.66)	59 (39.86)		
Yes	244 (35.11)	155 (28.34)	89 (60.14)		
Daily outdoor activity time, *n* (%)				χ^2^ = 102.53	<0.001
< 1 h	275 (39.57)	163 (29.80)	112 (75.68)		
≥ 1 h	420 (60.43)	384 (70.20)	36 (24.32)		
Daily mobile phone use time, *n* (%)				χ^2^ = 158.68	<0.001
0 h	375 (53.96)	362 (66.18)	13 (8.78)		
< 0.5 h	228 (32.81)	125 (22.85)	103 (69.59)		
≥ 0.5 h	92 (13.24)	60 (10.97)	32 (21.62)		
Daily television viewing time, *n* (%)				χ^2^ = 143.23	<0.001
0 h	366 (52.66)	352 (64.35)	14 (9.46)		
< 1 h	230 (33.09)	131 (23.95)	99 (66.89)		
≥ 1 h	99 (14.24)	64 (11.70)	35 (23.65)		
Daily after-school study time, *n* (%)				χ^2^ = 174.27	<0.001
0 h	138 (19.86)	52 (9.51)	86 (58.11)		
< 1 h	365 (52.52)	319 (58.32)	46 (31.08)		
≥ 1 h	192 (27.63)	176 (32.18)	16 (10.81)		
Nighttime sleep duration, *n* (%)				χ^2^ = 35.73	<0.001
< 7 h	181 (26.04)	120 (21.94)	61 (41.22)		
7–8 h	291 (41.87)	225 (41.13)	66 (44.59)		
> 8 h	223 (32.09)	202 (36.93)	21 (14.19)		
Daytime nap duration, *n* (%)				χ2 = 34.06	<0.001
<0.5 h	189 (27.19)	122 (22.30)	67 (45.27)		
0.5–1 h	275 (39.57)	223 (40.77)	52 (35.14)		
> 1 h	231 (33.24)	202 (36.93)	29 (19.59)		

### 3.2 Association between sleep duration and myopia

For daytime nap duration, both unadjusted and partially adjusted models (adjusting for demographic and family factors) showed a significant negative association with myopia. This inverse relationship remained after full adjustment (continuous: OR = 0.87, *p* = 0.012), with the 30–60 min and > 60 min groups showing 89 and 90% lower risks of myopia, respectively (both *p* < 0.05) (Table 2).

**Table 2:** Association between myopia and sleep duration.

Variables	Model 1		Model 2		Model 3	
	OR (95%CI)	*P*	OR (95%CI)	*P*	OR (95%CI)	*P*
Daytime nap duration continue	0.98 (0.97 ~ 0.99)	<0.001	0.96 (0.94 ~ 0.98)	<0.001	0.87 (0.77 ~ 0.97)	0.012
Daytime nap duration category						
< 30 min	1.00 (Reference)		1.00 (Reference)		1.00 (Reference)	
30–60 min	0.42 (0.28 ~ 0.65)	<0.001	0.18 (0.07 ~ 0.47)	<0.001	0.11 (0.02 ~ 0.49)	0.004
> 60 min	0.26 (0.16 ~ 0.43)	<0.001	0.07 (0.02 ~ 0.23)	<0.001	0.10 (0.02 ~ 0.59)	0.012
Nighttime sleep duration continue	0.70 (0.62 ~ 0.79)	<0.001	0.71 (0.57 ~ 0.89)	0.003	0.48 (0.25 ~ 0.94)	0.031
Nighttime sleep duration category						
< 7 h	1.00 (Reference)		1.00 (Reference)		1.00 (Reference)	
7–8 h	0.58 (0.38 ~ 0.87)	0.009	0.63 (0.27 ~ 1.51)	0.300	0.38 (0.04 ~ 4.11)	0.428
> 8 h	0.20 (0.12 ~ 0.35)	<0.001	0.17 (0.06 ~ 0.48)	<0.001	0.04 (0.00 ~ 0.94)	0.046

Model 1: Crude.

Model 2: Adjusted for: sex, age, BMI, grade, family income, marital status, paternal education level, maternal education level, residence, paternal myopia, maternal myopia.

Model 3: Adjusted for: sex, age, BMI, grade, family income, marital status, paternal education level, maternal education level, residence, paternal myopia, maternal myopia, daily outdoor activity time, daily mobile phone use time, daily television viewing time, daily after-school study time.

For nighttime sleep duration, the unadjusted model showed a significant negative association with myopia (continuous: OR = 0.70, *p* < 0.001). Compared with the < 7 h group, the 7–8 h and > 8 h groups had 42 and 80% lower risks of myopia, respectively (*p* = 0.009, *p* < 0.001). After adjusting for demographic and family factors, the negative association persisted for the continuous variable (OR = 0.71, *p* = 0.003) and the > 8 h group (OR = 0.17, *p* < 0.001), but the 7–8 h group was no longer significant (*p* = 0.300). With further adjustment for lifestyle factors, nighttime sleep duration remained negatively associated with myopia (continuous, per 1-h increment: OR = 0.48, *p* = 0.031), and the > 8 h group had a 96% lower risk of myopia (*p* = 0.046), while the 7–8 h group remained non-significant (*p* = 0.428) (Table 2).

### 3.3 RCS analysis and threshold effect analysis

**Figure 2:**
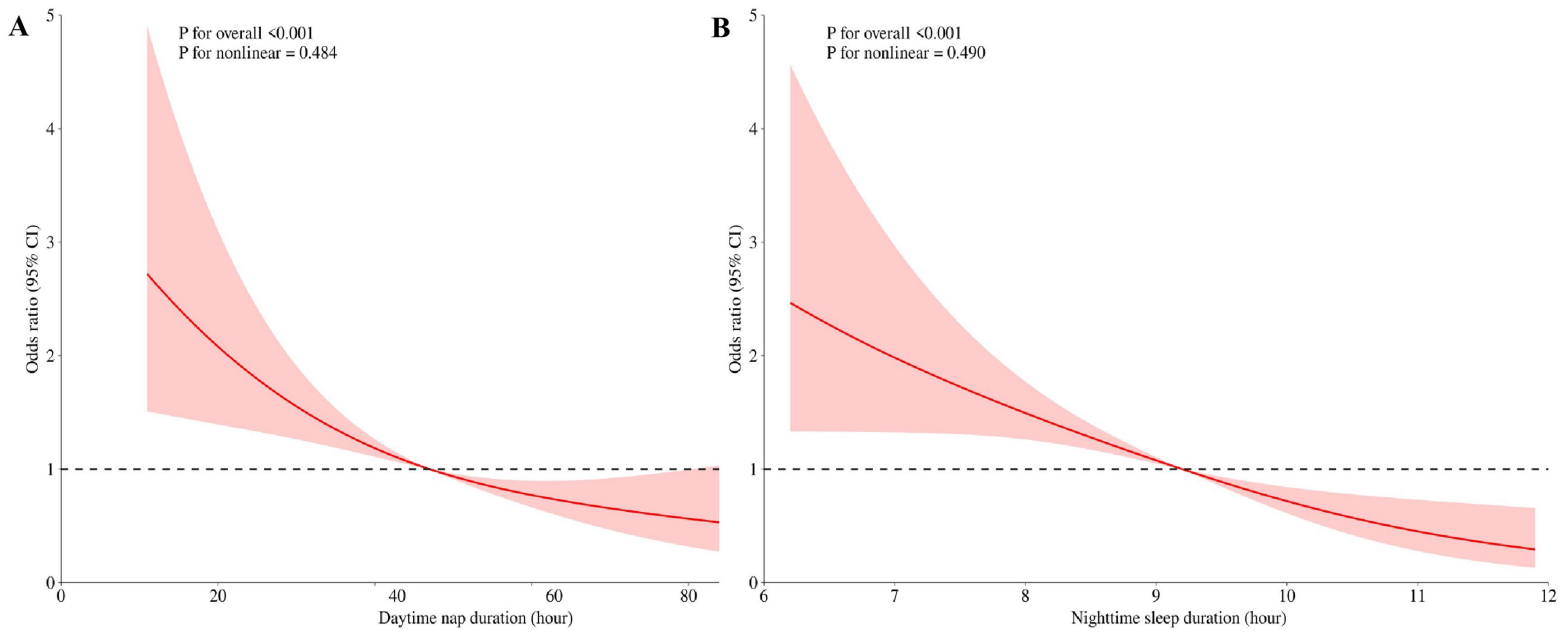
Restricted cubic spline analysis. **(A)** Daytime nap duration; **(B)** Nighttime sleep duration.

RCS curves showed a linear negative association between daytime nap duration and myopia risk (P-overall < 0.001, P-nonlinear = 0.484, Figure 2A). Threshold effect analysis identified an inflection point at 38 min: below this point, each 1-min increase in nap duration was associated with a 4% reduction in myopia risk (OR = 0.96, 95% CI: 0.93–1.00, *p* = 0.014); at or above the inflection point, each 1-min increase was associated with a 2% reduction (OR = 0.98, 95% CI: 0.97–1.00, *p* < 0.001). The inflection point was statistically significant (*p* < 0.001, Table 3). Nighttime sleep duration also showed a linear negative association (*P*-overall < 0.001, *P*-nonlinear = 0.390, Figure 2B), but no significant inflection point was detected (*p* = 0.652), and its protective effect remained stable across different sleep duration ranges (Table 3).

**Table 3:** Threshold effect analysis.

Outcome	Daytime nap duration			Nighttime sleep duration		
	Inflection point	Effect	*P*	Inflection point	Effect	*P*
Model 1 Fitting model by standard linear regression		0.98 (0.97–0.99)	<0.001		0.70 (0.62–0.79)	<0.001
Model 2 Fitting model by two-piecewise linear regression						
		38.00			9.32	
	<38.00	0.96 (0.93–1.00)	0.014	<9.32	0.76 (0.59–0.97)	0.026
	≥38.00	0.98 (0.97–1.00)	<0.001	≥9.32	0.60 (0.38–0.95)	0.029
*P* for likelihood test			<0.001			0.652

### 3.4 Subgroup analysis

**Figure 3:**
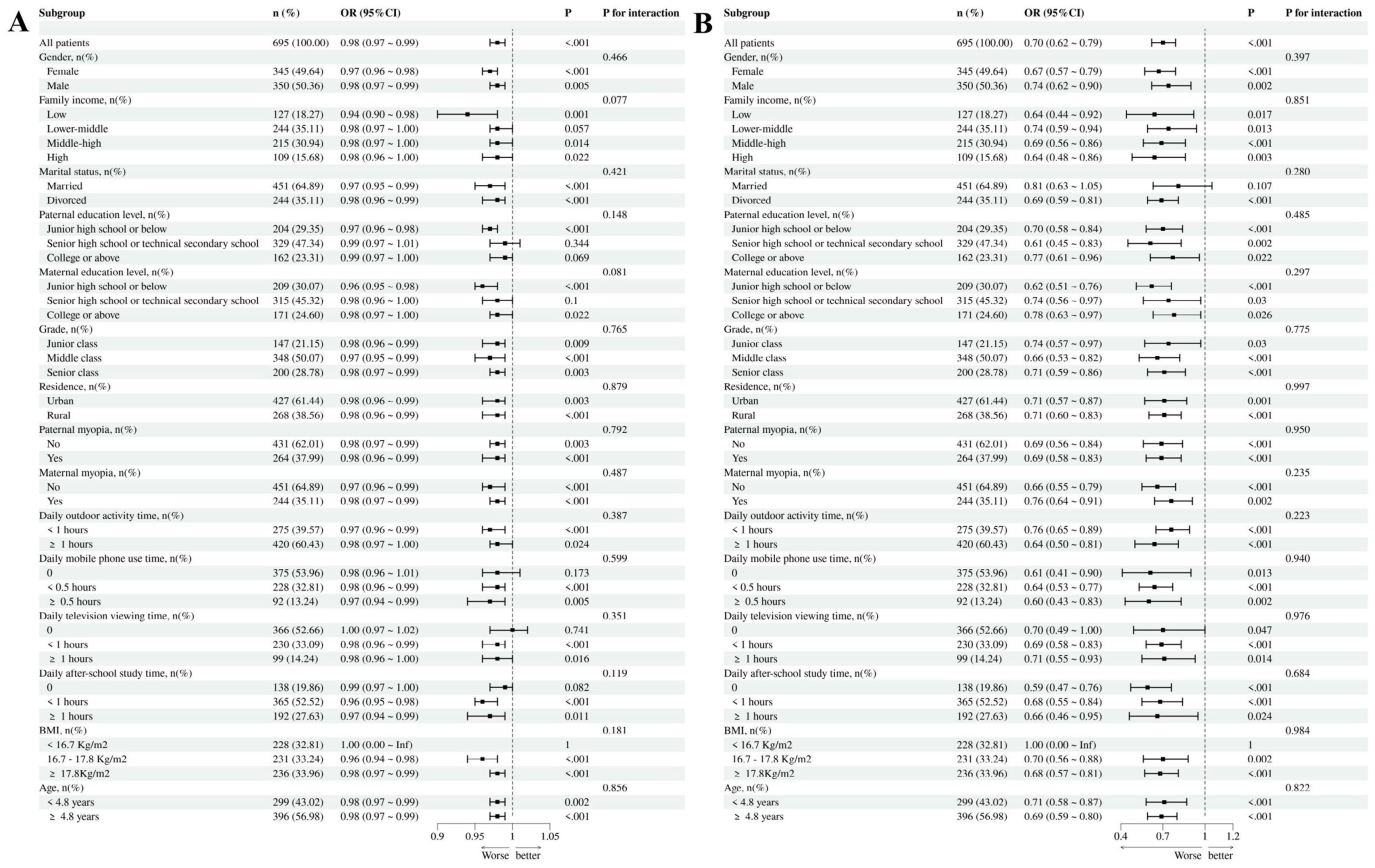
Forest plot of subgroup analysis. **(A)** Daytime nap duration; **(B)** Nighttime sleep duration.

Subgroup analysis showed that the inverse association between daytime nap duration and myopia remained consistent across different subgroups. After stratification by demographic, family, and lifestyle factors, the protective effect of nap duration was statistically significant in all subgroups (ORs ranging from 0.96 to 0.99, all *p* < 0.05), with no significant interactions observed (all *P* for interaction > 0.05), suggesting good consistency of this association across populations (Figure 3A).

**Figure 4:**
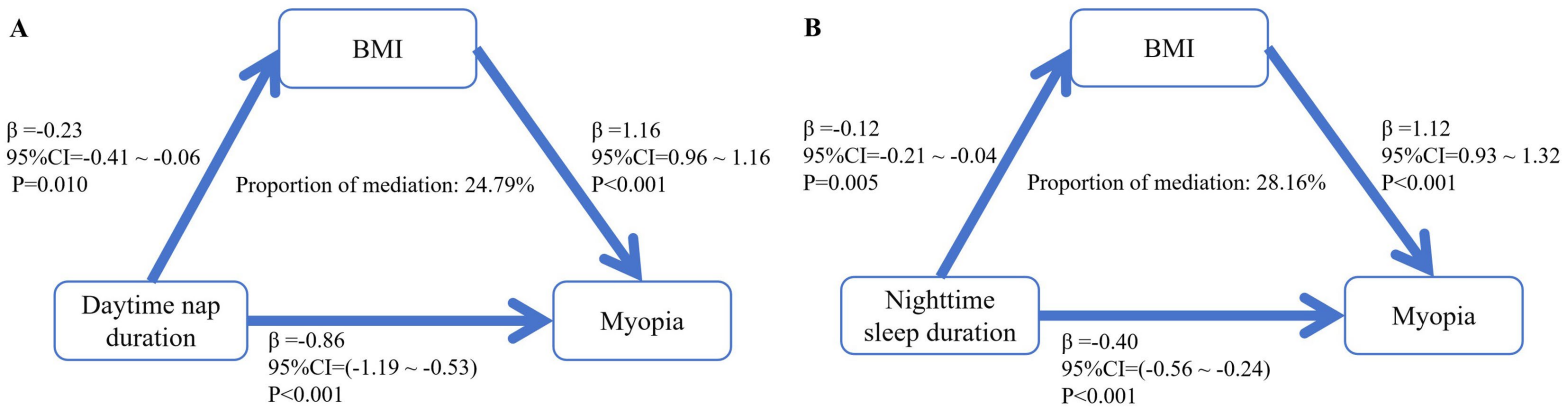
Mediation analysis of BMI in the association between sleep duration and myopia. **(A)** Daytime nap duration; **(B)** Nighttime sleep duration.

The inverse association between nighttime sleep duration and myopia was also consistent across most subgroups (Figure 3B). Notably, marital status showed an effect modification on this association (*P* for interactio*n* = 0.030). The protective effect was stronger in the divorced/separated group (OR = 0.69, 95% CI: 0.59–0.81), while it did not reach statistical significance in the married group (OR = 0.81, 95% CI: 0.63–1.05). No significant interactions were observed for other subgroup factors, indicating that the protective effect of nighttime sleep duration remained robust across those populations (Figure 4).

### 3.5 Mediation analysis

Mediation analysis showed that after full adjustment for demographic, family, and lifestyle factors, BMI played a mediating role in the associations of both daytime nap duration and nighttime sleep duration with myopia (Figures 2A,B). The ACME of BMI were −0.86 and −0.40, respectively (both *p* < 0.001), while the ADE were 0.23 and 0.12 (*p* = 0.010 and 0.005, respectively). The proportions of the total effect mediated by BMI were 24.79 and 28.16%, respectively.

## 4 Discussion

In this study, the prevalence of myopia among preschool children was 21.29%, a figure in line with that reported in Canadian children (28.9%) (19), but substantially higher than levels observed in Europe and Africa [1–4% in U.S. children aged 6–72 months (20), below 5% in European children aged 3–6 years (21), and 4.7% in African children and adolescents (22)]. Within China, prevalence estimates vary widely: 6.1% in a large Chengdu study of 108,578 children aged 3–6 year (23), 4.2% among kindergarten children in a recent meta-analysis (24). The higher prevalence in our sample (21.29%) may be attributed to hospital-based recruitment, a high proportion of children with parental myopia (37.99% of fathers and 35.11% of mothers), and geographic variations in myopia burden across China (24). These differences may be partly explained by the greater academic pressure and less time spent outdoors commonly seen in East Asian populations (16). Of note, surveillance data from Ningxia, China, between 1995 and 2019 showed that the prevalence of myopia among children and adolescents rose from 50.3 to 61.4%, a 10-percentage-point increase over 25 years (6). The prevalence of 21.29% in the present study was lower than the overall level observed in Ningxia, which is consistent with the younger age of our study population.

After adjusting for demographic characteristics, family factors, and lifestyle variables, both nighttime sleep duration and daytime nap duration were significantly and negatively associated with myopia risk, with evidence of a dose–response relationship. These findings are in line with those of several previous studies. A systematic review and dose–response meta-analysis including 45 observational studies and 766,848 children and adolescents aged 5–19 years found that longer sleep duration was associated with a 33% lower risk of myopia (OR = 0.67, 95% CI: 0.48–0.92 for the highest category) (8). A study from Ningxia, China, also reported that meeting the recommended sleep duration was an independent protective factor against myopia (OR = 0.722, 95% CI: 0.620–0.840, 6). A birth cohort study further showed that insufficient sleep at 5.5 years of age was significantly associated with increased axial length in school-aged children (*β* = 0.16, 95% CI: 0.07–0.24, 9). The French EDEN birth cohort study found a U-shaped association between sleep duration at 2 years of age and spectacle prescription at 5 years, with later bedtimes and later sleep midpoint also linked to a higher risk of prescription (10). In addition, the Korea National Health and Nutrition Examination Survey reported that each one-hour increase in sleep duration was associated with a 0.10 D increase in refractive error among adolescents aged 12–19 years (25). Collectively, this body of evidence suggests that the protective effect of sleep duration on myopia is generally consistent across different age groups (26). However, some inconsistency remains. A systematic review by Ding et al. pointed out that although longer sleep duration was significantly associated with a lower risk of myopia in categorical analyses, the protective effect did not reach statistical significance in the continuous dose–response analysis (8). Such discrepancies may be attributable to differences in sample age composition, sleep measurement methods, and adjustment for confounders across studies (27). By demonstrating a protective effect of sleep duration in preschool children, the present study suggests that the influence of sleep on myopia may already be evident at an earlier age. However, the association between sleep duration and myopia remains inconclusive. A recent dose–response meta-analysis found that while longer sleep duration was associated with lower myopia risk in categorical analyses, the protective effect did not reach statistical significance in continuous analysis (28). Another meta-analysis reported that short sleep duration was associated with increased myopia risk, while long sleep showed a protective effect (29). A Mendelian randomization study found no causal relationship between sleep traits and myopia, suggesting that observed associations may be confounded by other lifestyle factors (29). Several biological mechanisms may explain the sleep-myopia association. First, the dopamine-melatonin axis plays a central role: dopamine inhibits axial elongation and peaks during daytime, while melatonin facilitates circadian regulation of ocular growth at night. Sleep disruption alters this balance, weakening the retina’s natural brake against myopia progression (30). Second, sleep disturbance may reduce choroidal blood flow through sympathetic activation, leading to scleral hypoxia (31). Third, sleep disruption can trigger ocular inflammation, which may further accelerate myopia progression (31, 32). Fourth, insufficient sleep may reduce outdoor activity time, diminishing dopamine stimulation and attenuating the protective effect of light exposure (31). These pathways may interact synergistically to promote myopia development.

In this study, daytime nap duration showed a linear negative association with myopia, and threshold effect analysis identified 38 min as the inflection point for its protective effect. Few previous studies have specifically examined the association between napping and myopia. The Ma’anshan birth cohort study focused primarily on nighttime sleep rather than daytime naps, but it found that social jetlag of ≥1 h at age 4 was associated with an increased risk of myopia in school-aged children (9), suggesting that sleep regularity may play an important role in ocular health. Clinically, the 38-min inflection point suggests that a moderate daytime nap of around 38 min may optimally alleviate visual fatigue without disrupting nighttime sleep architecture, whereas shorter naps may be insufficient for ocular rest and longer naps may encroach upon nocturnal sleep or disrupt circadian homeostasis. Practically, this finding provides a quantifiable target for parental guidance and kindergarten scheduling, supporting structured nap periods of around 40 min. However, given that this is the first study to identify such an inflection point, our findings should be considered exploratory and require validation in future prospective studies. The inflection point identified in our study suggests that a moderate daytime nap of around 38 min may offer the greatest protection, possibly by alleviating visual fatigue and regulating circadian rhythms, whereas excessively long naps may compromise nighttime sleep quality and thereby weaken the protective effect (33).

Subgroup analysis revealed that the inverse association between nighttime sleep duration and myopia was more pronounced in divorced or separated families (OR = 0.69, 95% CI: 0.59–0.81), whereas it did not reach statistical significance in married families. This finding suggests that family environment may modulate the health effects of sleep, possibly by influencing sleep quality and regularity (34). Previous studies have shown that unstable family structures are associated with an increased risk of sleep disturbances in children (35); however, the underlying mechanisms of this interaction with myopia warrant further investigation.

Mediation analysis revealed that BMI partially mediated the associations of both daytime nap duration and nighttime sleep duration with myopia, accounting for 24.79 and 28.16% of the total effects, respectively. These findings suggest that BMI partly explains the protective effect of sleep duration on myopia, pointing to a potential role of metabolic factors in the biological pathways linking sleep to myopia. The biological plausibility of this mediating pathway is supported by several lines of evidence. First, insufficient sleep may promote weight gain through hormonal alterations. Short sleep duration has been shown to decrease leptin (an anorexigenic hormone) and increase ghrelin (an orexigenic hormone), leading to increased appetite and higher caloric intake, which subsequently contributes to elevated BMI (36). Second, obesity and myopia may be connected through multiple metabolic and inflammatory mechanisms. Adipose tissue in obese individuals secretes pro-inflammatory cytokines such as tumor necrosis factor-alpha and interleukin-6, which can induce systemic low-grade inflammation. This inflammatory state may affect scleral remodeling and axial elongation, key processes in myopia development (17). Additionally, insulin resistance commonly seen in obese children may stimulate insulin-like growth factor-1 signaling, which has been implicated in excessive ocular growth (18). Elevated intraocular pressure associated with higher BMI may also contribute to myopic changes through biomechanical effects on the sclera (37). Third, the circadian and metabolic systems are intimately linked; sleep disruption not only affects energy balance but also alters retinal dopamine metabolism and choroidal blood flow, pathways independently implicated in myopia control (31). The proportion of mediation was slightly higher for nighttime sleep duration than for daytime napping, which may reflect a more direct influence of nighttime sleep on the neuroendocrine regulation of metabolism. In summary, while BMI appears to serve as a partial mediator, the remaining direct effect of sleep on myopia likely operates through additional pathways, including circadian rhythm disruption and visual fatigue, which warrant further investigation.

This study has several limitations. In particular, the temporal sequence between sleep duration and myopia remains unclear. While we hypothesized that insufficient sleep may increase myopia risk, the reverse direction cannot be excluded. Myopia related visual discomfort such as asthenopia or accommodative spasm might disrupt sleep patterns in affected children (38). Future longitudinal studies are warranted to clarify the directionality of these associations. Second, sleep duration was assessed by parent-reported questionnaires rather than objective measures such as actigraphy or accelerometry. Although parent-reported questionnaires are the most practical and cost-effective method for large epidemiological studies, they are susceptible to recall and reporting bias. Parental reports may overestimate sleep duration, and nap duration accuracy may be particularly limited (39). Future studies incorporating objective sleep measurements would help validate our findings (40, 41). Third, the sample was drawn from a single hospital-based population using convenience sampling, which may limit the representativeness and generalizability of our findings to the broader preschool population. Multi-center studies with random sampling are needed to confirm the external validity of our results. Fourth, information on social jetlag and sleep regularity was not collected, although previous studies have shown these factors to be independently associated with myopia (42). Fifth, we did not collect data on sleep quality, bedtime, or wake time; cycloplegic refraction was not performed for all participants, which may have affected prevalence estimates; and residual confounding cannot be fully excluded despite extensive adjustment. Despite these limitations, our findings offer practical implications: clinicians should counsel parents on adequate sleep (≥8 h/night) and regular schedules, particularly for children with a family history of myopia; kindergartens should structure nap periods around 40 min based on the 38-min inflection point; and policymakers should integrate sleep health into myopia prevention frameworks. Additionally, promoting healthy weight may represent an additional preventive pathway given BMI’s partial mediating role.

## 5 Conclusion

In conclusion, both nighttime sleep duration and daytime nap duration were inversely associated with myopia risk in preschool children, with evidence of dose–response relationships. The protective effect of daytime napping showed an inflection point at 38 min, highlighting the importance of appropriate nap duration. BMI partially mediated the association between nighttime sleep duration and myopia, accounting for 28.16% of the total effect. These findings suggest that ensuring adequate nighttime sleep and appropriate daytime napping in preschool children may represent an effective strategy for early myopia prevention.

## Data Availability

The raw data supporting the conclusions of this article will be made available by the authors, without undue reservation.

## References

[1] Flitcroft DI He M Jonas JB Jong M Naidoo K Ohno-Matsui K . IMI - defining and classifying myopia: a proposed set of standards for clinical and epidemiologic studies. Invest Ophthalmol Vis Sci. (2019) 60:M20–30. doi: 10.1167/iovs.18-25957, 30817826 PMC6735818

[2] Holden BA Fricke TR Wilson DA Jong M Naidoo KS Sankaridurg P . Global prevalence of myopia and high myopia and temporal trends from 2000 through 2050. Ophthalmology. (2016) 123:1036–42. doi: 10.1016/j.ophtha.2016.01.006, 26875007

[3] Haarman AEG Enthoven CA Tideman JWL Tedja MS Verhoeven VJM Klaver CCW. The complications of myopia: a review and Meta-analysis. Invest Ophthalmol Vis Sci. (2020) 61:49. doi: 10.1167/iovs.61.4.49, 32347918 PMC7401976

[4] Tang M Liu Y Qin R Guo X Li H. Epidemiological characteristics of myopia and pre-myopia among preschool children aged 5-6 years in ten provinces of China. Beijing Da Xue Xue Bao. (2025) 57:442–7. doi: 10.19723/j.issn.1671-167X.2025.03.006 40509820 PMC12171601

[5] Morgan IG French AN Ashby RS Guo X Ding X He M . The epidemics of myopia: aetiology and prevention. Prog Retin Eye Res. (2018) 62:134–49. doi: 10.1016/j.preteyeres.2017.09.004, 28951126

[6] Cao J Xie X Li J Zhang L Chen Q Ma J . The prevalence of myopia and its association with sleep duration, physical activity, and eye exercises. Semin Ophthalmol. (2025) 40:815–22. doi: 10.1080/08820538.2025.2492256, 40249389

[7] Zhao X He Y Zhang J Lin S Zou H Ma Y. Effects of insufficient sleep on myopia in children: a systematic review and Meta-analysis. Nat Sci Sleep. (2024) 16:1387–406. doi: 10.2147/NSS.S472748, 39308665 PMC11416795

[8] Ding H Jiang L Lin X Ye C Chun B. Association of physical activity, sedentary behaviour, sleep and myopia in children and adolescents: a systematic review and dose-response meta-analysis. BMC Public Health. (2025) 25:1231. doi: 10.1186/s12889-025-22434-8, 40170130 PMC11959732

[9] Wang M Tong J Zhu D Huang K Wu X Gao G . Sleep duration, sleep habits, and social jetlag from 4 to 6 years their impacts on myopia among school-aged children: the Ma'anshan birth cohort study. Nat Sci Sleep. (2025) 17:365–78. doi: 10.2147/NSS.S500191, 40051711 PMC11883176

[10] Rayapoulle A Gronfier C Forhan A Heude B Charles MA Plancoulaine S. Longitudinal association between sleep features and refractive errors in preschoolers from the EDEN birth-cohort. Sci Rep. (2021) 11:9044. doi: 10.1038/s41598-021-88756-w, 33907290 PMC8079679

[11] Nickla DL Totonelly K. Brief light exposure at night disrupts the circadian rhythms in eye growth and choroidal thickness in chicks. Exp Eye Res. (2016) 146:189–95. doi: 10.1016/j.exer.2016.03.003, 26970497 PMC4893914

[12] Nickla DL. Ocular diurnal rhythms and eye growth regulation: where we are 50 years after Lauber. Exp Eye Res. (2013) 114:25–34. doi: 10.1016/j.exer.2012.12.013, 23298452 PMC3742730

[13] Jin E Lee CE Li H Tham YC Chen DZ. Association between sleep and myopia in children and adolescents: a systematic review and meta-analysis. Graefes Arch Clin Exp Ophthalmol. (2024) 262:2027–38. doi: 10.1007/s00417-023-06338-0, 38091060

[14] Fung V Price M Nierenberg AA Hsu J Newhouse JP Cook BL. Assessment of behavioral health services use among low-income Medicare beneficiaries after reductions in coinsurance fees. JAMA Netw Open. (2020) 3:e2019854. doi: 10.1001/jamanetworkopen.2020.19854, 33030552 PMC7545309

[15] Zhao W Wang J Chen J Xie H Yang J Liu K . The rate of orthokeratology lens use and associated factors in 33,280 children and adolescents with myopia: a cross-sectional study from Shanghai. Eye (Lond). (2023) 37:3263–70. doi: 10.1038/s41433-023-02503-1, 37046055 PMC10564736

[16] Morgan IG Ohno-Matsui K Saw SM. Myopia. Lancet. (2012) 379:1739–48. doi: 10.1016/S0140-6736(12)60272-4, 22559900

[17] Lee S Lee HJ Lee KG Kim J. Obesity and high myopia in children and adolescents: Korea National Health and nutrition examination survey. PLoS One. (2022) 17:e0265317. doi: 10.1371/journal.pone.0265317, 35333875 PMC8956184

[18] Cordain L Eaton SB Brand Miller J Lindeberg S Jensen C. An evolutionary analysis of the aetiology and pathogenesis of juvenile-onset myopia. Acta Ophthalmol Scand. (2002) 80:125–35. doi: 10.1034/j.1600-0420.2002.800203.x, 11952477

[19] Yang M Luensmann D Fonn D Woods J Jones D Gordon K . Myopia prevalence in Canadian school children: a pilot study. Eye (Lond). (2018) 32:1042–7. doi: 10.1038/s41433-018-0015-5, 29391573 PMC5997685

[20] Wen G Tarczy-Hornoch K McKean-Cowdin R Cotter SA Borchert M Lin J . Prevalence of myopia, hyperopia, and astigmatism in non-Hispanic white and Asian children: multi-ethnic pediatric eye disease study. Ophthalmology. (2013) 120:2109–16. doi: 10.1016/j.ophtha.2013.06.039, 23953098 PMC3902090

[21] Williams KM Kraphol E Yonova-Doing E Hysi PG Plomin R Hammond CJ. Early life factors for myopia in the British twins early development study. Br J Ophthalmol. (2019) 103:1078–84. doi: 10.1136/bjophthalmol-2018-312439, 30401676 PMC6661230

[22] Kobia-Acquah E Flitcroft DI Akowuah PK Lingham G Loughman J. Regional variations and temporal trends of childhood myopia prevalence in Africa: a systematic review and meta-analysis. Ophthalmic Physiol Opt. (2022) 42:1232–52. doi: 10.1111/opo.13035, 35959749 PMC9804554

[23] Mu J Zhang Z Wu X Chen S Geng H Duan J. Refraction and ocular biometric parameters in 3-to 6-year-old preschool children: a large-scale population-based study in Chengdu, China. BMC Ophthalmol. (2024) 24:207. doi: 10.1186/s12886-024-03467-w, 38711043 PMC11071229

[24] Gao H Ma J Liu Z Wang J Wang W Ye L. Prevalence of myopia in Chinese children and adolescents: a systematic review and meta-analysis. J Glob Health. (2026) 16:04056. doi: 10.7189/jogh.16.04056, 41855423 PMC13002174

[25] Jee D Morgan IG Kim EC. Inverse relationship between sleep duration and myopia. Acta Ophthalmol. (2016) 94:e204–10. doi: 10.1111/aos.12776, 26031352

[26] Baird PN Saw SM Lanca C Guggenheim JA Smith IEL Zhou X . Myopia. Nat Rev Dis Primers. (2020) 6:99. doi: 10.1038/s41572-020-00231-4, 33328468

[27] Grzybowski A Kanclerz P Tsubota K Lanca C Saw SM. A review on the epidemiology of myopia in school children worldwide. BMC Ophthalmol. (2020) 20:27. doi: 10.1186/s12886-019-1220-0, 31937276 PMC6961361

[28] Wang XX Liu X Lin Q Dong P Wei YB Liu JJ. Association between sleep duration, sleep quality, bedtime and myopia: a systematic review and meta-analysis. Clin Experiment Ophthalmol. (2023) 51:673–84. doi: 10.1111/ceo.14277, 37468126

[29] Dong XX Xie JY Li DL Dong Y Zhang XF Lanca C . Association of sleep traits with myopia in children and adolescents: a meta-analysis and Mendelian randomization study. Prev Med. (2024) 180:107893. doi: 10.1016/j.ypmed.2024.107893, 38342383

[30] Chakraborty R Ostrin LA Nickla DL Iuvone PM Pardue MT Stone RA. Circadian rhythms, refractive development, and myopia. Ophthalmic Physiol Opt. (2018) 38:217–45. doi: 10.1111/opo.12453, 29691928 PMC6038122

[31] Liu S Zhou X Zhao J. How sleep disturbance promotes myopia: a perspective on potential biological mechanisms. Exp Eye Res. (2025) 261:110645. doi: 10.1016/j.exer.2025.110645, 40972857

[32] Zhang N Han G Hao R. The role of circadian rhythms in the pathogenesis of myopia. Front Physiol. (2026) 17:1797489. doi: 10.3389/fphys.2026.1797489, 41994054 PMC13078985

[33] Matricciani L Paquet C Galland B Short M Olds T. Children's sleep and health: a meta-review. Sleep Med Rev. (2019) 46:136–50. doi: 10.1016/j.smrv.2019.04.011, 31121414

[34] El-Sheikh M Kelly RJ. Family functioning and children's sleep. Child Dev Perspect. (2017) 11:264–9. doi: 10.1111/cdep.12243, 29731807 PMC5931738

[35] Gao Z Guo Z Song Y Shi X Zhao Y Liu C. Gender difference of the association between sleep duration and myopia among children and adolescents. Nat Sci Sleep. (2024) 16:1303–12. doi: 10.2147/NSS.S476051, 39247908 PMC11379028

[36] Taheri S Lin L Austin D Young T Mignot E. Short sleep duration is associated with reduced leptin, elevated ghrelin, and increased body mass index. PLoS Med. (2004) 1:e62. doi: 10.1371/journal.pmed.0010062, 15602591 PMC535701

[37] Han X Yang T Zhang J Yu S Guo X Yan W . Longitudinal changes in intraocular pressure and association with systemic factors and refractive error: Lingtou eye cohort study. BMJ Open. (2018) 8:e019416. doi: 10.1136/bmjopen-2017-019416, 29444785 PMC5829881

[38] Cai T Zhao L Kong L Du X. Complex interplay between COVID-19 lockdown and myopic progression. Front Med. (2022) 9:853293. doi: 10.3389/fmed.2022.853293, 35386915 PMC8978626

[39] Werner H Molinari L Guyer C Jenni OG. Agreement rates between actigraphy, diary, and questionnaire for children's sleep patterns. Arch Pediatr Adolesc Med. (2008) 162:350–8. doi: 10.1001/archpedi.162.4.350, 18391144

[40] Matricciani L Olds T Petkov J. In search of lost sleep: secular trends in the sleep time of school-aged children and adolescents. Sleep Med Rev. (2012) 16:203–11. doi: 10.1016/j.smrv.2011.03.005, 21612957

[41] Duarte A Martins J Rosario R. Agreement between parental reports and accelerometer measures of sleep duration in primary school children. Sci Rep. (2025) 15:25654. doi: 10.1038/s41598-025-07786-w, 40664721 PMC12263886

[42] Liu XN Naduvilath TJ Wang J Xiong S He X Xu X . Publisher correction: sleeping late is a risk factor for myopia development amongst school-aged children in China. Sci Rep. (2021) 11:4881. doi: 10.1038/s41598-021-84377-5, 33623099 PMC7902659

