# Supplementary material for: Cross-sectional associations of nighttime sleep and daytime nap duration with myopia in preschool children: the mediating role of body mass index
Source: Front Med (Lausanne). 2026 Jul 7;13:1840892. doi: 10.3389/fmed.2026.1840892 (PMC13386222; doi:10.3389/fmed.2026.1840892)
Supplement: Supplementary file 1 [file Data_Sheet_1.docx]

# Questionnaire on Sleep, Lifestyle, and Myopia in Preschool Children

Dear Parent/Guardian,

This questionnaire aims to understand your child's sleep habits, lifestyle, and vision status. All information will be used solely for scientific research and will be kept strictly confidential. Please answer based on your actual circumstances. Thank you for your cooperation!

Date of completion: ________________

Child's initials (first letter of each given name): ________________

## Part 1: Demographic Information

- **A1. Sex:**  □ Male □ Female
- **A2. Age:**  ______ years
- **A3. Grade:**  □ Junior class □ Middle class □ Senior class
- **A4. Height:**  ______ cm
- **A5. Weight:**  ______ kg

## Part 2: Family Factors

- **B1. Household registration:**  □ Urban □ Rural
- **B2. Monthly household income:**  □ <5,000 CNY □ 5,000–10,000 CNY □ 10,001–15,000 CNY □ >15,000 CNY
- **B3. Parental marital status:**  □ Married □ Divorced
- **B4. Father's education level:**  □ Junior high school or below □ Senior high school/vocational school □ College degree or above
- **B5. Mother's education level:**  □ Junior high school or below □ Senior high school/vocational school □ College degree or above
- **B6. Is the father myopic?:**  □ Yes □ No
- **B7. Is the mother myopic?:**  □ Yes □ No

## Part 3: Sleep Duration

- **C1. Weekday nighttime sleep duration:**  ______ hours
- **C2. Weekend nighttime sleep duration:**  ______ hours
- **C3. Daytime nap duration:**  ______ minutes

## Part 4: Lifestyle and Visual Behaviors

- **D1. Average daily outdoor activity time:**  □ <1 hour □ 1–2 hours □ 2–3 hours □ >3 hours
- **D2. Average daily mobile phone use time:**  □ 0 hours □ <0.5 hours □ ≥0.5 hours
- **D3. Average daily television viewing time:**  □ 0 hours □ <1 hour □ ≥1 hour
- **D4. Average daily after-school study time:**  □ 0 hours □ <1 hour □ ≥1 hour

— End of Questionnaire. Thank you for your participation! —
